# Assessment of progesterone profiles and postpartum onset of luteal activity in spring calving Hereford beef suckler cattle

**DOI:** 10.1186/1751-0147-52-42

**Published:** 2010-06-15

**Authors:** Adam D Martin, Marit L Lystad, Olav Reksen, Erik Ropstad, Andres Waldmann, Ola Nafstad, Knut Karlberg

**Affiliations:** 1 Department of Production Animal Clinical Sciences, Norwegian School of Veterinary Science, Oslo, Norway; 2 Animalia - Meat and Poultry Research Centre, Oslo, Norway; 3 Institute of Veterinary Medicine and Animal Sciences, Estonian University of Life Sciences, Tartu, Estonia

## Abstract

**Background:**

Reproduction is the single greatest factor limiting beef cattle production. Previous research on beef suckler luteal activity has largely focused on the mechanisms, and duration, of postpartum anoestrus. However, the temporal pattern of luteal activity after resumption of post-partum ovarian activity, and the impact of pattern type on days open (DO) in purebred beef suckler cows, are unknown.

**Methods:**

Progesterone concentration was measured in milk samples taken thrice weekly from 120 lactations, in 87 animals, on 3 farms, over two years. Onset of luteal activity (OLA) was defined as the first day milk progesterone concentration exceeded 3 ng/ml for two successive measurements, or exceeded 5 ng/ml once. It was defined as delayed if it occurred more than 61 days postpartum. A short initial luteal phase consisted of progesterone concentrations which exceeded 3 ng/ml for fewer than 4 sequential measurements. Temporal progesterone patterns were classified as: 1) Normal cyclicity; 2) Cessation of luteal activity; 3) Prolonged luteal activity; 4) Erratic phase: failure to conform to 1, 2 or 3. Data concerning parity, previous calving interval, breeding values, calf birth and 200-d weight were obtained from the Norwegian Beef Cattle Recording System database.

**Results:**

The mean (SD) OLA was 41 d (20). Parity and calf birth weight were inversely correlated with OLA. Delayed OLA occurred in 14.4% of lactations. A short first luteal phase occurred in 61.5% of lactations, but this was unrelated to irregular luteal phase occurrence, pregnancy or DO. Irregular luteal phases occurred in 22% of lactations. The irregularities were: prolonged luteal phase (11%); cessation of luteal activity (5%); erratic luteal activity (6%). Early OLA was associated with prolonged luteal phases. DO was positively correlated with irregular luteal phases and negatively correlated with calf 200-d weight.

**Conclusions:**

This study demonstrates that irregular luteal phases negatively affect reproductive performance in purebred beef suckler cattle. A moderate incidence of irregular luteal phases was seen in the study population. Whilst a positive relationship was seen between OLA and DO, unfavourable associations between early OLA and incidence of irregular luteal phases should be considered when developing breeding programmes.

## Background

Efficiency of beef production is of critical importance as the global demand for meat increases [1]. Furthermore, policy decisions taken at a European level in recent decades have encouraged lower stocking densities and a reduction in the use of nitrogen based fertiliser [2-4]. In Norway suckled calf production systems are largely pasture based, and despite recent increases in beef suckler cow numbers, the country is not self sufficient in beef production, with a net import of 10,700 tonnes of beef and beef products in 2008 [5]. It is, therefore, important to identify and understand the bottlenecks present in the production system if action is to be taken to improve production efficiency.

The greatest factor limiting suckled calf production efficiency is suboptimal reproductive performance [6,7]. The duration of postpartum anoestrus largely determines the probability of females becoming pregnant during the breeding season [7]. Considerable attention has been paid to the pattern of reproductive cyclicity in the dairy population, how it has changed with time, and the negative impacts of irregular luteal phases on reproductive performance [8-11]. Additional studies describe the risk factors for ovarian dysfunction in high yielding dairy cows [12,13]. The heritability of onset of luteal activity (OLA) is relatively high when compared with the heritability of traditional measures of fertility [14] and, to an increasing extent, its use in breeding programmes to improve fertility has been advocated [15-17].

A number of studies have investigated the time to OLA in beef cows and the factors influencing it [18]. However, to the authors' knowledge no studies have characterised the temporal pattern of luteal activity after OLA, factors influencing the pattern of luteal activity, or the impact of pattern type on days open (DO) in purebred beef suckler cows. It is possible that, in an attempt to improve production efficiency, beef breeding programmes will increasingly emphasise fertility. Therefore, it is imperative that the temporal pattern of luteal activity is characterised, factors influencing it assessed, and consequences evaluated, as this information may prove fundamentally important in the development of future breeding programmes.

The objectives of the current study were, in purebred, pasture grazed, late winter/spring calving, Hereford suckler cows to: i) characterise the postpartum OLA and subsequent pattern of luteal activity; ii) study the relationship between individual cow/calf variables (parity, maternal breeding value, calf birth weight, calf gender, calf 200 d weight) and OLA, pattern of luteal activity and DO.

## Methods

### Animals

The study was undertaken over a period of two years and included 120 lactations (26 first, 20 second, and 74 third or later lactations ) from 87 purebred Hereford cattle. In the first year, two farms were included in the study (Farm A, n = 28 animals and Farm B, n = 23 animals), a third farm (Farm C) was recruited in the second year (Farm A, n = 33 animals, Farm B, n = 18 animals and Farm C, n = 18 animals). No animals moved between the farms during the study. The three spring-calving commercial farms were all members of the Norwegian Beef Cattle Recording System (NBCRS) and volunteered to participate in the study. The reproductive management and feeding practices were similar on all three farms; concentrates, minerals and round-bale grass silage were fed during winter. Minerals and round bale silage supplemented the permanent pasture diet during the grazing season. Animals were separated from the herd for parturition, and rejoined the herd within three days of calving. Throughout the study, bulls, which had passed a breeding soundness examination, were kept with each herd at a female:male ratio below 40:1. Consequently, females were exposed to a bull constantly from Day 3, or earlier, post-partum. Pregnancy diagnosis was performed by transrectal palpation of the uterus every 3 weeks to identify 6 to 9 week pregnancies. The study was performed within the guidelines of the Norwegian School of Veterinary Science's Ethical committee.

### Sampling and Assay of Milk Progesterone

Milk samples were collected thrice weekly from ten days postpartum until pregnancy had been confirmed. Samples were frozen within one hour of collection and transported to the hormone laboratory at the Norwegian School of Veterinary Science. Progesterone concentrations were determined from whole milk by enzyme immunoassay [19], using the second antibody coating technique [20]. The inter-assay coefficients of variation for progesterone concentration in whole milk at progesterone concentrations of 1.5 and 19.7 ng/ml were 9.2 and 5.3%, respectively. The intra-assay coefficient of variation progesterone concentration in whole milk was less than 10%. The limit of sensitivity, using a 20 μl sample, was less than 0.5 ng/ml.

### Onset of Luteal Activity

The day of OLA after calving was determined using milk progesterone concentration data. It was defined as the first day that milk progesterone concentrations were greater than 3 ng/ml for two successive measurements, or greater than 5 ng/ml once [19,21]. Onset of luteal activity was defined as normal if it occurred within one standard deviation of the study population mean; early if it occurred before this, and delayed if it occurred after this.

### Temporal Pattern of Progesterone Concentration

The initial luteal phase was described as short if progesterone concentrations exceeding 3 ng/ml were maintained for fewer than 4 measurements. Animals that experienced a short first luteal phase had their temporal pattern of progesterone concentration classified without this first, short, cycle. The temporal patterns of progesterone concentration were classified using categories described by Mann et al. [22]:

1) **Normal cyclicity**: periods of progesterone concentration below 3 ng/ml for less than 1 week followed by progesterone concentrations exceeding 3 ng/ml for more than 2 weeks, or high levels of progesterone concentration (exceeding 3 ng/ml) in association with confirmed pregnancy.

2) **Cessation of luteal activity**: progesterone concentration less than 3 ng/ml for more than 2 weeks following a period of luteal activity.

3) **Prolonged luteal activity**: progesterone concentration greater than 3 ng/ml for more than 3 weeks, in the absence of pregnancy.

4) **Erratic phase**: failure to conform with 1, 2 or 3.

Lactations with normal cyclicity were regarded as having regular luteal phases, whilst those which were not classified as having normal cyclicity were regarded as having irregular luteal phases.

### Individual Cow-Calf Variables

Data on parity, previous calving interval, breeding values, calf birth weight and calf 200-d weights were obtained from the NBCRS database. Days open (DO) was defined as the number of days from calving until the last milk sample with a progesterone concentration measurement below 3 ng/ml preceding confirmed pregnancy was taken. Maternal calf 200-d weight breeding values were calculated using a best linear unbiased prediction (BLUP) model by the Norwegian Beef Breeding Association http://www.tyr.no. Maternal calf 200-d weight breeding value and calf 200-d weight were both tested as continuous variables and categorised by their quartiles. Four cows gave birth to twins: their data were removed from the statistical analyses.

### Statistical Analyses

The likelihood of luteal phase irregularities was assessed using the categories 'regular' and 'irregular', in separate analyses the likelihood of 'prolonged luteal activity' was compared with 'normal cyclicity'. Only significant results from the 'prolonged luteal activity' vs. 'normal cyclicity' analyses have been reported. The dichotomous outcome variables were tested against explanatory variables both univariately and in multivariable models using a general estimating equation (GEE) approach with the GENMOD procedure in SAS [23]. Animals were nested within herd, which was accounted for by entering farm as a fixed effect to all analyses. Overall statistical significance was assessed by the score statistics for type III GEE Analysis.

Onset of luteal activity, DO, and previous calving interval were transformed using their natural logarithm because of the non-normality of these data, and tested as continuous variables. Relationships between the continuous outcome variables; lnOLA and lnDO; and the explanatory variables were tested both univariately and in multivariable models using Proc Mixed in SAS [24]. Measurements between lactations within farms were not independent; 'farm' was included as a random factor in the model. Overall statistical significance was assessed by the type III F-test.

The inclusion of two lactations from individual animals in this study was accounted for by using a first order autoregressive correlation structure in all models. In all analyses statistical significance was considered with a *P*-value below 0.05. Explanatory variables with a *P*-value below 0.20 in the univariate analyses were simultaneously entered in a multivariable model together with the first order interaction between all variables. A backwards elimination procedure was employed and variables with a *P*-value below 0.10 were retained in the model. Confounding was assessed by comparing parameter estimates. If the estimates varied by more than 20%, confounding was regarded as being present [25].

## Results

In seven lactations accurate determination of OLA was not possible, and these lactations were omitted from the analyses. Consequently, accurate assessment of the time from calving until to OLA was possible in 113 lactations (25 first, 17 second, and 71 third or later lactations) in 87 individual cows. The mean interval from calving until OLA (SD) was 41 d (20). Thus normal OLA was defined as being between 21 and 61 d. Early OLA was seen in ten lactations (9%). Delayed OLA occurred in sixteen lactations (14%).

Sufficient data were available to make an accurate assessment of the length of the first luteal phase in 108 out of 113 lactations (24 first, 17 second, and 67 third or later lactations). Once luteal activity had begun a short luteal phase occurred in 63 of 108 lactations (Farm A 32/57, Farm B18/36, Farm C 13/15). Short first luteal phases were seen more frequently on Farm C than on Farms A and B combined (*P *= 0.02). The odds ratio for a short first luteal phase was predicted to be 0.74 and 4.83 in Farms B (n = 36) and C (n = 15), respectively compared to Farm A (n = 57) (*P *= 0.02). No associations were seen between the presence of a short first luteal phase and likelihood of pregnancy (*P *= 0.42), lnDO (*P *= 0.14) or time from lnOLA to pregnancy (*P *= 0.77).

Univariate analyses of the relationships between lnOLA and the explanatory variables can be seen in Table 1. Parity was inversely related to lnOLA when assessed univariately (*P *< 0.01), and primiparous animals took longer to OLA than both second lactation and third or later lactation cows (47, 42 and 32 days respectively). An inverse univariate relationship (*P *= 0.001) was observed between lnOLA and calf birth weight; the increase of calf birth weight by 1 kg (range 24 to 57 kg) shortened lnOLA by 0.04 units (range 3.08 to 4.26). The model estimated OLA to be 40 days in a cow giving birth to a calf of 40 kg body weight; whereas time to OLA in a cow giving birth to a calf of 50 kg was predicted to be 28 days. Calf birth weights differed (*P *< 0.05) with calf gender: bull calves weighed on average 43 kg at birth (n = 56) and heifer calves 41 kg (n = 51).

**Table 1:** Univariate relationships between the natural logarithm of onset of luteal activity and study variables

Variable	Group	n	β-value	S.E.	F-value	*P*-value
Calf 200-d weight		85	0.001	0.002	0.09	0.77
Calf 200-d weight	1^st ^quartile	20	0.049	0.156	0.39	0.76
	2^nd ^quartile	24	0.148	0.143		
	3^rd ^quartile	20	0.073	0.142		
	4^th ^quartile	21	0.000	0.000		
Calf birth weight		95	-0.036	0.011	11.49	0.001
Calf birth weight	1^st ^quartile	21	0.337	0.132	3.80	0.01
	2^nd ^quartile	27	0.354	0.127		
	3^rd ^quartile	21	0.050	0.118		
	4^th ^quartile	24	0.000	0.000		
Calf gender	Male	53	0.057	0.084	0.46	0.49
	Female	55	0.000	0.000		
Natural logarithm of previous calving interval		85	0.098	0.387	0.06	0.80
Maternal breeding value		102	0.006	0.006	1.07	0.30
Maternal breeding value	1^st ^quartile	26	-0.026	0.151	0.02	1.00
	2^nd ^quartile	31	-0.023	0.145		
	3^rd ^quartile	22	-0.040	0.162		
	4^th ^quartile	23	0.000	0.000		
Parity	1^st^	25	0.377	0.113	5.84	<0.01
	2^nd^	17	0.210	0.117		
	>2^nd^	70	0.000	0.000		

When multivariable relationships were assessed between lnOLA and the explanatory variables parity and calf birth weight, only the latter remained significantly (*P *= 0.02) associated with lnOLA. After correction for the effect of parity, the predicted decrease in lnOLA per kg increase in calf birth weight changed from 0.036 to 0.028, which indicates that parity and birth weight are confounding variables [25].

The temporal pattern of progesterone concentration could not be categorized in four lactations due to missing data. Thus the classification of luteal activity was performed in 116 lactations (26 first, 19 second, and 71 third or later lactations). Irregular luteal phases, i.e. lactations not classified as having normal cyclicity, occurred in 26 of 116 (22.4%) lactations. The irregularities characterised were: prolonged luteal phase (n = 13; 11.2%), cessation of luteal activity (n = 6; 5.2%), and erratic phase (n = 7; 6.0%). The results of univariate analyses of the relationships between the explanatory variables and incidence of irregular luteal phases are given in Table 2. Irregular luteal phases tended to be related to both lnOLA (*P *= 0.06) and categorized calf birth weight (*P *= 0.06). However, when analysed in the multivariable model, only categorised calf birth weight remained after the application of the backwards elimination procedure, reducing the multivariable model to a univariate assessment.

**Table 2:** Univariate relationship between incidence of irregular luteal phases and study variables

Variable	Group	N	β-value	SE	Odds ratio	Chi-square	*P*-value
Calf 200-d weight		88	0.008	0.006	1.01	1.25	0.27
Calf 200-d weight	1^st ^quartile	22	-0.646	0.643	0.52	1.75	0.63
	2^nd ^quartile	24	0.296	0.664	1.34		
	3^rd ^quartile	20	-0.131	0.787	0.88		
	4^th ^quartile	22	0.000	0.000	1.00		
Calf birth weight		98	0.069	0.063	1.07	1.35	0.25
Calf birth weight	1^st ^quartile	21	-0.280	0.706	0.76	7.48	0.06
	2^nd ^quartile	29	-1.983	0.948	0.14		
	3^rd ^quartile	23	0.003	0.598	1.00		
	4^th ^quartile	25	0.000	0.000	1.00		
Calf gender	Male	56	0.115	0.437	1.12	0.07	0.79
	Female	56	0.000	0.000	1.00		
Natural logarithm of onset of luteal activity		110	-1.003	0.537	0.37	3.58	0.06
Natural logarithm of previous calving interval		89	2.220	2.697	0.11	0.77	0.38
Maternal breeding value		107	0.032	0.026	1.03	1.45	0.23
Maternal breeding value	1^st ^quartile	26	-1.674	0.751	0.19	6.50	0.08
	2^nd ^quartile	33	-0.001	0.550	1.00		
	3^rd ^quartile	22	-0.969	0.691	0.38		
	4^th ^quartile	26	0.000	0.000	1.00		
Parity	1^st^	26	-0.443	0.619	0.64	1.20	0.55
	2^nd^	19	0.475	0.613	1.61		
	>2^nd^	71	0.000	0.000	1.00		

When the likelihood of prolonged luteal phases was assessed separately, the odds ratio of a prolonged luteal phase occurring was 5.33 for each unit decrease in lnOLA (range: 2.30 to 4.68) (*P *< 0.01). This indicates a strong likelihood of a prolonged luteal phase occurring in cows with early OLA.

In total eleven animals were not identified as becoming pregnant in either one of the annual breeding seasons. The mean (SD) DO in pregnant animals was 73 d (33) (n = 109). Pregnancy was established earlier than Day 40 in 16 (14.6%) lactations, and later than Day 106 in 16 (14.6%) lactations.

Table 3 displays the univariate analysis of lnDO and the explanatory variables. Parity was negatively associated with lnDO; first parity animals were predicted to be pregnant on Day 80, second parity on Day 58, third or later parity on Day 64 postpartum. Luteal phase irregularities, both overall and as assessed solely for prolonged luteal phases, were positively related to an increased number of DO (*P <*0.01). The model predicted a 25 d increase in DO in those animals experiencing irregular luteal phases. The time until OLA was positively associated with DO. Maternal 200-d calf weight breeding value and calf 200-d weight were negatively associated with DO.

**Table 3:** Univariate relationships between natural logarithm of days open and studied variables

Variable	Group	n	β-value	SE	F-value	*P*-value
Calf 200-d weight		81	-0.003	0.001	5.49	0.02
Calf 200-d weight	1^st ^quartile	21	0.286	0.100	3.14	0.03
	2^nd ^quartile	22	0.151	0.099		
	3^rd ^quartile	19	0.250	0.113		
	4^th ^quartile	19	0.000	0.000		
Calf birth weight		91	-0.008	0.010	0.56	0.46
Calf birth weight	1^st ^quartile	20	0.034	0.129	1.83	0.15
	2^nd ^quartile	27	-0.206	0.114		
	3^rd ^quartile	23	-0.070	0.125		
	4^th ^quartile	21	0.000	0.000		
Calf gender	Male	51	-0.005	0.080	0.00	0.95
	Female	54	0.000	0.000		
Luteal phase pattern	Normal	84	-0.342	0.085	16.34	0.001
	Irregular	24	0.000	0.000		
Luteal phase pattern	Other	96	-0.290	0.114	6.42	0.01
	Prolonged	12	0.000	0.000		
Natural logarithm of onset of luteal activity		103	0.162	0.081	3.96	0.05
Natural logarithm of previous calving interval		78	-0.452	0.440	1.06	0.31
Maternal breeding value		100	-0.009	0.004	5.09	0.02
Maternal breeding value	1^st ^quartile	24	0.267	0.096	3.10	0.03
	2^nd ^quartile	32	0.221	0.086		
	3^rd ^quartile	21	0.188	0.091		
	4^th ^quartile	23	0.000	0.000		
Parity	1^st^	23	0.231	0.095	3.82	0.03
	2^nd^	19	-0.101	0.106		
	>2^nd^	67	0.000	0.000		

When multivariable relationships were assessed between lnDO and the explanatory variables, the variables luteal phase pattern, lnOLA, calf 200-d weight, calf birth weight categorised by its quartiles and parity remained in the model after the backwards selection procedure had been employed (Table 4). Compared to second parity cows and third or later parity cows, the model predicted that primiparous animals would experience 24 more DO. Animals that experienced luteal phase irregularities took 26 days longer to become pregnant than animals with normal cyclical activity. After correction for the explanatory variables, which included luteal phase irregularities, the multivariable model indicated that lnDO increased by 0.26 units per unit increase in lnOLA.

**Table 4:** Multiple relationships between natural logarithm of days open and explanatory variables

Variable	Group	n	β-value	SE	F-value	*P*-value
Calf 200-d weight		81	-0.003	0.001	11.25	0.001
Calf birth weight	1^st ^quartile	21	-0.356	0.127	4.25	0.01
	2^nd ^quartile	22	-0.285	0.085		
	3^rd ^quartile	19	-0.224	0.100		
	4^th ^quartile	19	0.000	0.000		
Luteal phase pattern	Normal	84	-0.370	0.089	17.19	<0.001
	Irregular	24	0.000	0.000		
Natural logarithm of onset of luteal activity		103	0.263	0.067	15.21	<0.001
Parity	1^st^	23	0.261	0.097	3.73	0.03
	2^nd^	19	0.001	0.097		
	>2^nd^	67	0.000	0.000		

## Discussion

The time from calving to OLA was predictably longer in the study population than has been recorded in dairy cattle [8-11,26,27]. The time from calving to OLA in the present study falls in the middle of the range (29 to 67 d) provided by the review of 23 studies on beef suckler animals between 1963 and 1999 [18]. The relationship between parity and time to OLA, with primiparous animals taking a longer time to OLA than pluriparous cows, is well established [18,27,28]. The proportion of beef suckler cows exhibiting delayed and early OLA in this study was similar to a study involving beef cross dairy animals and those involving dairy cows [8-11,22,27].

Onset of luteal activity was positively associated with DO in the present study. When analysed in a univariate model parity, but not lnOLA, was related to DO. However, when analysed in a more sophisticated multivariable model both parity and lnOLA, along with calf 200-d weight, were related to DO. This is probably because parity is closely related to lnOLA. The parameter estimates for lnOLA changes by almost 25% between the univariate and multivariable models, indicating confounding between the variables [25]. Taken together, DO was positively related to OLA, when the effect of parity was accounted for.

In this study the majority (61%) of ovarian activity began with a short luteal phase, which concurs with previous studies [29-31]. Progesterone concentrations postpartum can be raised by the luteinization of ovarian follicles, or more commonly, after ovulation, with formation of a corpus luteum [32]. Short luteal phases are known to occur with an increased frequency in anoestrous suckler cows after the weaning of their calves [33]. In our study the risk of short luteal phases was higher on one of the farms compared to the other two, indicating that factors other than suckling are involved in determining their frequency.

The importance of short luteal phases for normal luteal cyclicity and oestrus expression has been discussed [31,34]. Ciccioli et al. [31] found that no cow displayed normal oestrous behaviour before the first postpartum progesterone concentration rise, but all cows did after this transient increase. Whilst Looper et al. [34] found that 81% of luteal phases preceded by a short luteal phase were normal, compared to just 36% that were not. However, in the present study no association was seen between the presence, or absence, of a short luteal phase at OLA and the likelihood of pregnancy or DO, and pregnancy coincided with the first progesterone concentration rise in 11 animals (10%). Consequently, short luteal phases were not a prerequisite of normal reproductive function for the beef cattle in this study.

In the present study, 22% of lactations were associated with irregular luteal phases, considerably higher than the 7% incidence previously reported in beef cross dairy animals by Mann et al. [22]. The occurrence of irregular luteal phases increased DO in this study; this agrees with previous studies on dairy cattle [8-10,27]. The incidence of irregular luteal phases excluding delayed OLA, reported in the present study is similar to modern dairy populations (between 13 and 44%) [8,10,11,27,35]. The increased incidence of irregular luteal phases found in dairy cattle are believed to be the result of intensive selection and management to produce high milk yields [8,10,27]. However, the selection and management pressures applied to the Hereford breed have been, and are, very different to those experienced by dairy cattle. This indicates that a certain level of irregular luteal phases may be regarded as normal in beef cattle as well as in dairy cows.

The incidence of prolonged luteal phases in this study was far greater than in a previous study of beef cross dairy animals, 11% compared to 3% [22]. Early OLA is a risk factor for the occurrence of prolonged luteal phases in dairy cows [10-12]. Onset of luteal activity generally occurs earlier in dairy cows than in beef cows [18]. Therefore, it is interesting that in this study early OLA, as defined for beef cows, was associated with an increased incidence of prolonged luteal phases despite a number of the known risk factors (high milk yields, intensive genetic selection for milk production and uterine disease) being absent.

A possible confounding factor in this study was the use of unrecorded natural service. Previous estimates for late embryonic/early fetal loss range between 3 and approximately 10% [9,22,36]. Whilst the current study indicates an association between early OLA and prolonged luteal phases further research is necessary to quantify the relative impact of embryonic/early fetal loss on this finding. However, as natural service is predominant in the beef suckler systems [37] the practical significance of this finding remains important. The current study implies that there may be an optimum time to introduce fertile bulls to cows postpartum to maximise reproductive performance as embryonic/early fetal loss will also increase DO. Furthermore, as unfavourable associations exist between early OLA and incidence of irregular luteal phases mean that the inclusion of early OLA in breeding programmes should proceed cautiously, at least until the effects of early OLA on the pattern of luteal activity are better understood.

Heavier calves at birth, when analysed in the multivariable model, were associated with an increased number of DO. However, in multivariable model predicting lnOLA, which accounted for the effects of parity, higher calf birth weights *per se *were associated with an earlier OLA in the current study. Heavier calves are more likely to be associated with dystocia and postpartum uterine disease, both of which are known to delay OLA [38,39]. Dystocia did not occur in the study population allowing a positive association between fetal growth rate and OLA to be revealed. This relationship may have been masked in previous studies by the relationship between high birth weight and dystocia.

Interestingly, 200-d calf weight breeding value and calf 200-d weight were negatively associated with DO. This is reverse to the findings in previous studies which show increasing milk yields to be associated with increased calf weaning weight, and a decrease in subcutaneous fat depth, indicating negative energy balance [40,41], which in turn may adversely affect reproductive performance [18]. However, reported correlations between milk yield and calf 200-d weight vary considerably in previous studies (between 0 and 0.8) [42], and further work is needed to explain the observed relationship.

## Conclusion

This study demonstrates a moderate incidence of irregular luteal phases in purebred beef suckler cattle compared to previous studies in dairy cattle. The occurrence of a short luteal phase immediately following OLA did not influence the likelihood of abnormal luteal phases, likelihood of pregnancy or DO under the study conditions. Generally OLA was positively associated with DO. However, unfavourable associations were seen between early OLA and incidence of irregular luteal phases. Irregular luteal phases were shown to negatively affect reproductive performance, as measured by DO. This relationship should be considered when developing future breeding programmes.

## Competing interests

The authors declare that they have no competing interests.

## Authors' contributions

AM prepared the manuscript. AM, ML, OR, ER, ON, and KK were involved in the planning of the study. ER coordinated the laboratory analysis. AW prepared and supplied the progesterone assay used. AM and OR decided upon and performed the statistical analysis. All authors have read and approved the manuscript.
